# Regional diagnostic panels for aeroallergens in Canada

**DOI:** 10.1186/1710-1492-10-S1-A17

**Published:** 2014-03-03

**Authors:** Joshua Young, Robert Erskine, Tricia Moore, Greg Plunkett

**Affiliations:** 1 ALK, Inc. Round Rock, TX, USA

## Background

Prevalence of allergenic plants varies by geographic region and climate, which affects the level of allergen exposure experienced by patients in different parts of the country. Because of these variations in exposure it is recommended that allergy practices use customized regional diagnostic panels based on the prevalence and significance of various aeroallergens. However, gathering this information can be difficult for new physicians. The purpose of these recommendations is to provide a foundation for new physicians to begin building a custom aeroallergen panel for all regions of Canada.

## Methods

Clinical, geographical and botanical references were evaluated and compiled to determine the prevalence and impact of various aeroallergens across Canada. These recommendations were discussed with regional allergy practices and other clinical authorities for consensus on recommendations.

## Results

Aeroallergen recommendations were compiled into a prevalence map and table that was organized by the major geographic regions of Canada. Allergens were categorized as (1) high allergenicity & high prevalence, (2) high allergenicity & low prevalence, (3) low allergenicity & high prevalence, or (4) low allergenicity & low prevalence. A significant degree of allergen similarity across all regions was recognized although specific differences in species selection and general distribution patterns were identified for each region.

## Conclusions

Gathering clinical and botanical prevalence data for aeroallergens across different regions can be time consuming and difficult. These recommendations were compiled from many years of industry experience working with allergy specialists across the country and were verified by the literature. It is hoped that this knowledge can provide a foundation for new physicians trying to understand which aeroallergens to target for allergy diagnostic panels specific to their region.

